# A Rare Atypical Case of a Pulled Elbow in an Infant

**DOI:** 10.7759/cureus.74689

**Published:** 2024-11-28

**Authors:** Syarul Arwiz Sahruzaman, Norazian Kamisan

**Affiliations:** 1 Orthopaedics, University of Putra Malaysia, Serdang, MYS

**Keywords:** nursemaid's elbow, orthopaedics & traumatology, paediatric elbow injuries, paediatric orthopedics, pulled elbow

## Abstract

A pulled elbow is a common type of injury in children aged one to four years, where the forearm is pulled in an extended pronated position. There are a few cases of pulled elbow reported in children under one year old. We experienced an atypical pulled elbow case in a six-month-old girl after her mother rolled her from a right lateral position to a supine position, leaving her arm trapped behind her back. The pulled elbow was suspected based on the classical presentation of pain over the elbow, a less mobilized limb, and a pronated arm position as well as the exclusion of fracture from a plain radiograph. A high index of suspicion of pulled elbow should be kept in infants, despite their age presentation and the absence of an obvious elbow-pulling mechanism.

## Introduction

Pulled elbow, also known as nursemaid’s elbow, is a subluxation of the radial head from the annular ligament caused by sudden longitudinal traction or pulling of the forearm in an extended, pronated position [1]. This condition most commonly occurs in children aged one to four years [2]. We experienced an atypical case of pulled elbow in a six-month-old girl after her mother rolled her from a right lateral position to a supine position, leaving her arm trapped behind her back. After the incident, the mother noticed that the child was crying and refusing to move her right upper limb. A plain radiograph revealed no fractures and failed to exclude subluxation of the radial head. Clinical suspicion of a pulled elbow was made due to tenderness in the right elbow, forearm in a pronated position, and the child's refusal of active or passive flexion of the right elbow. A high index of suspicion for a pulled elbow should be maintained in infants, despite their atypical age presentation and the absence of an obvious elbow pulling mechanism. Prompt reduction helps to reduce the radial head and gain normal function.

## Case presentation

A six-month-old girl with no known medical conditions was brought by her mother to the emergency department after the child was crying and refused to move her right upper limb. There was no trauma, fall, or pulling action toward the right upper limb preceding the incident. The mother claimed she had rolled her daughter during sleep from her right lateral position into a supine position, leaving her right arm trapped behind her back, followed by crying and an inability to move her right upper limb. This was her first occurrence, and suspicion of a non-accidental injury has been excluded.

**Figure 1:**
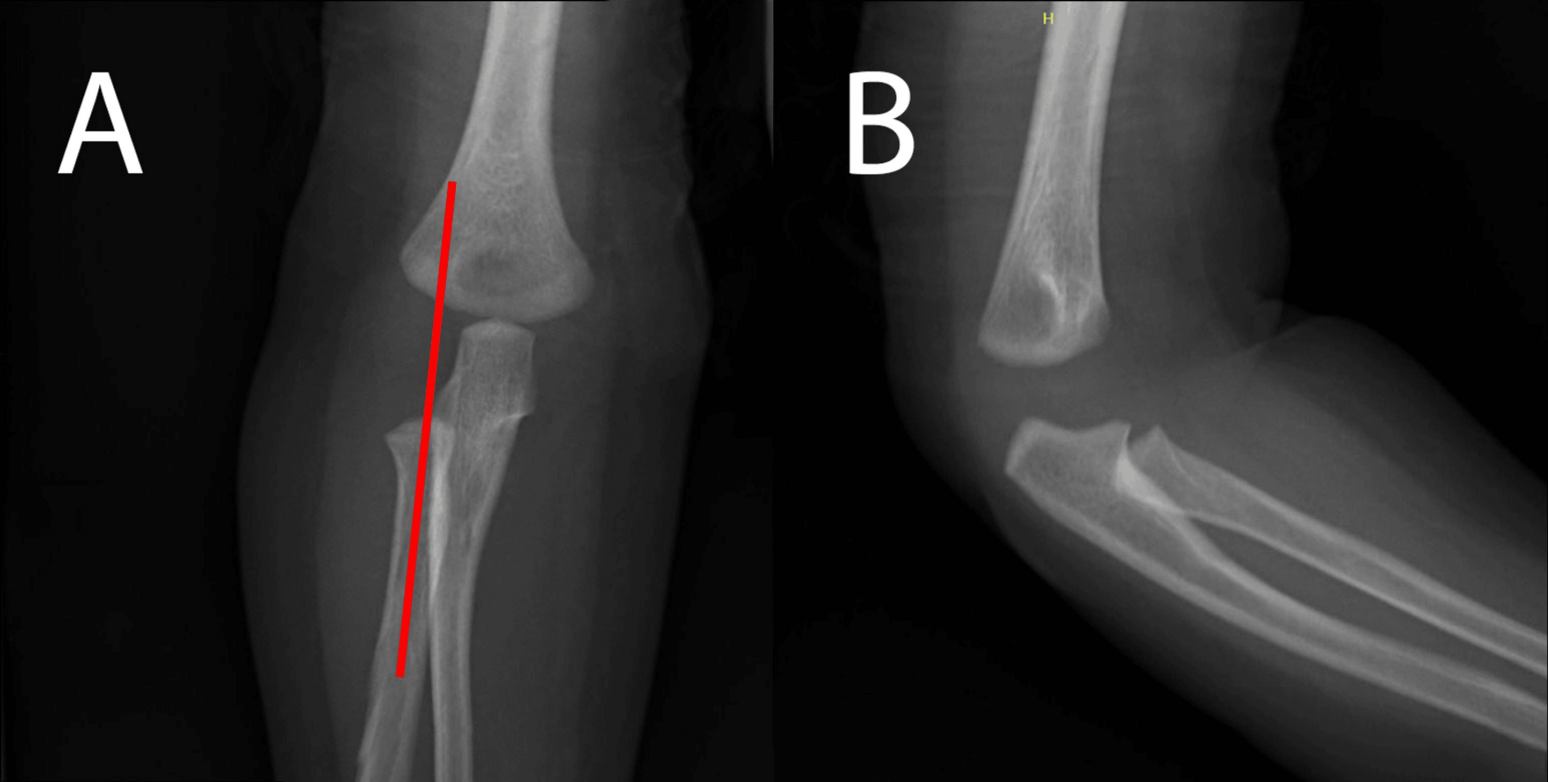
Plain radiograph of the right elbow: No signs of fracture are present. The radiocapitellar line appears normal (red line). Anterior-posterior view (A) and lateral view (B) of right elbow plain radiographs. The radiocapitellar line is marked by a red line in Panel A.

On examination, the child was calm, lying supine on the bed, with no signs of bruises on the head, body, or rest of the limbs. The child’s right upper limb is in an extended pronated position and appears less mobilized compared to the left upper limb. However, no obvious swelling or deformity was noted. The right elbow was tender on palpation and appeared hesitant and crying upon passive movement of the elbow. A plain radiograph of the right upper limb revealed no fracture, and the x-ray of the right elbow appears normal (Figure 1). A pulled elbow was suspected based on clinical assessment and findings. After explaining to the mother and consenting to a trial of reduction, closed manipulation reduction was performed in the emergency department with adequate analgesia (syrup Ibuprofen 5 mg/kg). A supination-flexion method was carried out while pressure was applied over the radial head. A palpable clunk was felt during the maneuver on the first attempt. Post-reduction, the child appears more comfortable and starts mobilizing her right upper limb. The child was observed for a few hours before being safely discharged. Before discharge, no tenderness was elicited on the right elbow, as the child does not hesitate in the active and passive movements of the elbow and has a full range of motion.

## Discussion

Pulled elbows commonly occur in older children, around one to four years of age [2]. They are rare in infants under one year old, with only a few cases reported [3]. Pulled elbows occur mostly due to shallow anatomical, concave radial head, which is cartilaginous in nature, and the immature annular ligaments. The mechanism of injury is a longitudinal pulling of the arm in an extended pronated position, which occurs more commonly in active and weight-bearing children. Plain radiographs are recommended to exclude fractures [4].

Pulled elbow is a diagnosis of exclusion after ruling out fractures of the elbow (supracondylar humerus, condylar, olecranon, and radial head fracture), septic arthritis elbow, and congenital radial head dislocation or forearm synostosis.

A high index of suspicion of a pulled elbow in children needs to be kept in mind when an infant presents with the tenderness of the elbow, refuses to use the affected limb, and has a pronated forearm attitude.

Most plain radiographs of the elbow may show insignificant findings (subluxation or dislocation of the radial head) and are indistinguishable from healthy elbow radiographs [5]. Whenever in doubt, an ultrasound of the elbow may help in confirming the diagnosis with the presence of a J-shaped hypoechoic image between the capitellum and radial head with a sensitivity and specificity of 100% [6].

The pulled elbow should be attempted to be reduced via closed manipulation, either via supination-flexion technique (forearm in supination position, gradual flexion of the elbow, and gentle pressure over the radial head) or hyperpronation technique (flex elbow with hyperpronation of the forearm). Both methods are effective, though the reduction of pulled elbows with the hyperpronation method has a higher success rate of 93.84% on the first attempt [7]. Surgical intervention is rarely required in pulled elbow cases. A successful reduction will hear a click sound or be felt as the radial head is reduced. The child should begin to mobilize the limb immediately after a successful reduction of the radial head. Immobilization of the reduced pulled elbow in the first encounter case is rarely needed.

As preventive measures, the pulled elbows can be prevented by always lifting them by supporting their underarms instead of pulling their hands or wrists. Avoid swinging them by their arms or hands to prevent stress on their elbow joints, and engage in gentle play that does not involve pulling or tugging on their arms.

## Conclusions

A high index of suspicion for a pulled elbow is necessary in infants presenting with pain over the elbow, a less mobilized limb, and a pronated position, after ruling out other possible causes. Prompt reduction helps to reduce the radial head back into position, subsequently reducing the pain and gaining back elbow function.
